# Obstructive lung disease and quality of life after cure of multi-drug-resistant tuberculosis in Uganda: a cross-sectional study

**DOI:** 10.1186/s41182-020-00221-y

**Published:** 2020-05-19

**Authors:** Edwin Nuwagira, Anna Stadelman, Joseph Baruch Baluku, Joshua Rhein, Pauline Byakika-Kibwika, Harriet Mayanja, Ken M. Kunisaki

**Affiliations:** 1 Department of Medicine, Mbarara University of Science and Technology, P.O Box 1410, Mbarara, Uganda; 2 Division of Infectious Diseases and International Medicine, Department of Medicine, University of Minnesota, Minneapolis, MN USA; 3 Division of Epidemiology and Community Health, School of Public Health, University of Minnesota, Minneapolis, MN USA; 4 Department of Medicine, Makerere University College of Health Sciences, Kampala, Uganda; 5 Section of Pulmonary, Critical Care and Sleep Medicine, Minneapolis VA Health Care System, Minneapolis, MN USA; 6 Division of Pulmonology, Allergy, Critical Care and Sleep Medicine, Department of Medicine, University of Minnesota, Minneapolis, MN USA

**Keywords:** Multi-drug-resistant tuberculosis, HIV, Uganda, COPD, Quality of life

## Abstract

**Background:**

Pulmonary multi-drug-resistant tuberculosis (MDR TB) alters lung architecture and involves lengthy treatment duration, high pill burden, drug adverse effects, travel restrictions, and stigma. Literature about pulmonary function and health-related quality of life (QoL) of patients treated for MDR TB is limited. This study sought to determine the prevalence of chronic obstructive pulmonary disease (COPD) and QoL of patients who were treated for pulmonary MDR TB.

**Methods:**

Participants who completed 18 months of pulmonary MDR TB treatment and considered cured were eligible to be evaluated in a cross-sectional study. We performed post-bronchodilator spirometry to measure forced expiratory volume in 1 s (FEV_1_) and forced vital capacity (FVC). COPD was defined as FEV_1_/FVC < 0.7; health-related QoL was assessed using the Medical Outcomes Survey for HIV (MOS-HIV) and St. George’s Respiratory Questionnaire (SGRQ). Linear and logistic regression models were used to assess associations with COPD, health-related QoL, and other characteristics of the cohort.

**Results:**

A total of 95 participants were enrolled. Median age of the cohort was 39 years (interquartile range (IQR), 29–45), and 55 (58%) were HIV-positive. COPD prevalence was 23% (22/95). Median SGRQ score was normal at 7.8 (IQR, 3.1–14.8). Median mental and physical health summary scores were significantly impaired, at 58.6 (IQR, 52.0–61.5) and 52.9 (IQR, 47.8–57.9), respectively, on a scale of 0 to 100 where 100 represents excellent physical or mental health. In this sample, 19% (18/95) of participants were in the lowest relative socioeconomic position (SEP) while 34% (32/95) were in the highest relative SEP. Belonging in the lowest SEP group was the strongest predictor of COPD.

**Conclusion:**

Individuals who have completed MDR TB treatment have a high prevalence of COPD and low mental and physical health summary scores. Our study highlights the need for pulmonary rehabilitation programs in patients with a low socioeconomic position (SEP) after MDR TB treatment.

## Background

The global prevalence of pulmonary multi-drug-resistant tuberculosis (MDR TB) continues to rise and is of major global concern, with approximately half a million people infected worldwide with MDR/rifampicin resistant (RR) tuberculosis in 2018 alone [1]. MDR TB disease destroys the lung architecture, characterized by bronchiectasis, pleural disease, and reduced lung volume [2, 3]. It is therefore not surprising that as high as 98% of patients completing MDR TB treatment have impaired lung function [2]. Impaired lung function can be obstructive or restrictive. Our study was only focused on obstructive function. In Uganda, the prevalence of COPD among adults aged 30–39 years is approximately 16%, with biomass and tobacco use being the major risk factors [4]. With this known, the association between chronic obstructive pulmonary disease (COPD) and MDR TB has not been elucidated.

The standard treatment of MDR TB is lengthy with a significant pill burden and long hospitalization. MDR TB patients report depression, stigma, discrimination, side effects of the drugs, inability to work, social isolation, and financial constraints [5–7]. These factors most likely affect health-related quality of life (QoL) of individuals that have completed MDR TB treatment.

The World Health Organization’s (WHO) End TB Strategy focuses on reducing TB deaths, incident TB cases, and household catastrophic costs due to TB [1]. However, none of these strategies considers long-term end-organ complications of MDR TB such as COPD or the psychosocial impact of the disease. Currently, there is a paucity of recommendations for assessing QoL or lung function after MDR TB treatment, and studies are needed to inform the formulation of guidelines to address this [8]. We aimed to determine the prevalence of COPD and assess the quality of life of patients after completing pulmonary MDR TB treatment at two large teaching hospitals in Uganda.

## Methods

### Study aim

The aim of the study was to determine the prevalence of COPD and assess the QoL of patients after completion of MDR TB treatment in Uganda.

### Study population

Between January 2018 and April 2018, we used a cross-sectional study design to enroll 95 participants that had completed MDR TB treatment at Mulago National Referral Hospital or Mbarara Regional Referral Hospital in Uganda. Potential participants were identified from the MDR TB registers at each study site and consecutively called. Those who were ≥ 18 years of age, completed MDR TB treatment, and declared cured were contacted using the mobile telephone number listed in the registry and screened for eligibility. Potential participants were screened for the following exclusion criteria: extrapulmonary tuberculosis and known contraindications to post-bronchodilator spirometry testing such as documented active tuberculosis, pneumothorax, known allergy to Albuterol, known cardiac pathology, and ocular symptoms suggestive of retinal detachment. We consecutively called the 138 potential participants of whom 95 reported to the study sites. All 95 participants were consented and considered for the study.

### Study setting

We conducted the study at two tertiary teaching hospitals in Uganda: Mulago National Referral Hospital and Mbarara Regional Referral Hospital. Mulago National Referral Hospital’s TB treatment unit is located in the capital city of Kampala, Uganda. Mbarara Regional Referral Hospital is a rural hospital located in the western region of Uganda, 265 km from the capital. These two have the highest patient enrollment of 18 MDR TB treatment sites in the country.

### Study measurements

#### Pulmonary function

At both sites, we measured spirometry using standardized equipment (Vitalograph® Pneumotrac, Model 6800, Ennis, Ireland), which met equipment and procedure requirements of the American Thoracic Society/European Respiratory Society (ATS/ERS) spirometry standards [9]. A certified respiratory physiologist performed pre-bronchodilator spirometry on all participants and a post-bronchodilator spirometry on all those meeting criteria for obstruction by the American Thoracic guidelines. The spirometers had been factory calibrated before use in our study, and the technicians checked daily calibration with a 3-L syringe. We were interested in the lung function (forced expiratory volume in 1 s (FEV_1_)) and the forced vital capacity (FVC). We included two acceptable trials if the best values were within 200 mL of one another and participants who had a ratio of FEV_1_ to FVC less than 0.7 were subjected to a post-bronchodilator test using four puffs (360 μg) of Albuterol (Ventolin, GSK, Philadelphia PA). Spirometry was then repeated after 10 min. COPD was defined as a ratio of FEV_1_ to FVC < 0.7 [10].

#### Surveys of health-related QoL and socioeconomic position

The Saint George’s Respiratory Questionnaire (SGRQ), Medical Outcomes Survey for HIV (MOS-HIV), and socioeconomic position (SEP) survey were translated into the two different local languages spoken at the two research sites. Study staff from both sites were trained on how to administer the surveys to participants through a face-to-face interview before spirometry.

The SGRQ measures respiratory health status. It has 50 items with 76 weighted responses and is scored from 0 to 100, where the higher score indicates worse respiratory health status [11]. The MOS-HIV is a comprehensive assessment of HIV/AIDS-specific QoL. The MOS-HIV has 30 items and is scored from 0–100 and provides both physical health summary (PHS) and mental health summary (MHS) scores, where the higher score indicates better health status. We used a culturally adapted MOS-HIV that has been previously validated among patients with TB in Uganda [12, 13]. SEP survey is a relative wealth index for Uganda available from www.equitytool.org/uganda/. The relative wealth index uses possession of household items to measure the relative wealth of those in the sample. Those with the highest relative wealth were placed in the fifth position, while those with the lowest relative wealth were placed in the first position. Those in the first SEP were considered living in poverty. The SEP questionnaire was modified to capture data on employment status, body mass index (as measured by study assistant), time since MDR TB treatment completion, education level, and smoking status.

### Statistical analysis

Due to the exploratory nature of this study, the sample size was not calculated. Data were entered and stored in a web-based data analysis application, REDCap (Vanderbilt University, Nashville, NT, USA). Statistical analyses were carried out in SAS (Cary, NC, USA) version 9.4. Descriptive statistics of the participants, as well as the results of the lung function testing and QoL assessments, are reported as medians with interquartile ranges (IQR). Statistical significance tests included *t* tests, nonparametric tests, or chi-square tests as appropriate. The Global Lung Function Initiative 2012 equations were used as reference values for calculating percent predicted and less than lower limit of normal (LLN) values for this cohort [14]. We used bivariate logistic regression models to assess the association of age, sex, time since MDR TB treatment completion (months), any history of smoking, HIV status, MHS scores, PHS scores, SGRQ score, and poverty status (defined as those in the first SEP) with COPD. If a *p* value was 0.25 or smaller, the variable was included in the multivariate logistic regression model.

## Results

A total of 240 patients identified on the MDR TB registers were alive after treatment. Of these, 27 had been assigned “completed treatment” as a treatment outcome and 213 were declared cured between January 2011 and December 2017. Of these potential participants, 138 had active telephone numbers. We consecutively called the 138 potential participants of whom 95 reported to the study sites. Upon physical exam, all 95 participants were eligible for the study.

The baseline characteristics of the participants are described in Table 1. Median age was 39 (IQR 29–45) years, 55 (58%) were HIV-positive, and 57 (60%) were male. There were no notable characteristic differences between the HIV-positive and HIV-negative groups (Table 1).

**Table 1:** Baseline characteristics of study population

	Median (IQR) or *N* (%)
Age, years	39 (29, 45)
Male	57 (60%)
Education	
Partial or complete tertiary	14 (15%)
Partial or complete secondary	24 (25%)
Partial or complete primary	48 (51%)
No formal education	9 (9%)
Employed	57 (60%)
Months since completion of MDR TB treatment	20 (9, 28)
BMI, kg/m^2^	20.2 (18.3, 22.8)
Smoking status	
Current	1 (1%)
Former	23 (24%)
Never	71 (75%)
HIV-positive	55 (58%)
MOS-HIV	
Physical health summary score	58.6 (52.0, 61.5)
Mental health summary score	52.9 (47.8, 57.9)
SGRQ	
Total score	7.8 (3.1, 14.8)
Median score for those with FEV_1_ ≥ 80% of predicted	4.9 (3.1, 10.5)
Median score for those with FEV_1_ 50–79% of predicted	9.4 (3.1, 19.4)
Median score for those with FEV_1_ < 49% of predicted	14.2 (9.4, 16.8)
SEP	
1—poorest	18 (19%)
2	10 (11%)
3	15 (16%)
4	20 (21%)
5—wealthiest	32 (34%)

*MOS-HIV* Medical Outcomes Survey HIV, *SGRQ* St. George’s Respiratory Questionnaire, *SEP* socioeconomic position

### Lung function and COPD prevalence

Median FEV_1_ was 2.1 L (IQR 1.7–2.6) and 74.9% of predicted normal (IQR 62.4–88.9). Median FEV_1_/FVC ratio was 0.8 (IQR 0.7–0.8), and the prevalence of COPD was 23% (22/95) (Table 2). In a multivariable model, COPD was associated with poverty (OR = 3.12 95% CI (0.87, 11.7); *p* = 0.08) but not HIV status, time since treatment completion, or smoking history (Table 3). A post hoc analysis using an alternate COPD definition of FEV_1_/FVC < LLN is shown in Additional file 1.

**Table 2:** Spirometry results and COPD (*n*= 95)

Measure of lung function	Median (IQR) or *N* (%)
FEV_1_, L	2.1 (1.7, 2.6)
FEV_1_ ≥ 80% of predicted	36 (38%)
FEV_1_ 50–79% of predicted	47 (49%)
FEV_1_ 30–49% of predicted	12 (13%)
FEV_1_ < 30% of predicted	0 (0%)
FEV_1_ percent predicted	74.9 (62.4, 88.9)
FVC, L	2.9 (2.4, 3.3)
FVC percent predicted	81.9 (69.0, 89.8)
FEV_1_/FVC ratio	0.78 (0.70, 0.83)
FEV_1_/FVC ratio percent predicted	93.5 (84.1, 101.7)
FEV_1_/FVC < LLN	29 (31%)
FEV_1_/FVC < 0.70 (COPD)	22 (23%)
FEV_1_ ≥ 80% of predicted	0 (0%)
FEV_1_ 50–79% of predicted	12 (55%)
FEV_1_ 30–49% of predicted	10 (45%)
FEV_1_ < 30% of predicted	0 (0%)

*COPD* chronic obstructive pulmonary disease, *LLN* lower limit of normal

**Table 3:** Multivariate logistic regression parameter estimates for COPD

Outcome	Odds ratios (95% confidence interval)	*p* value
COPD		
Age, per 5 years	1.24 (0.92, 1.67)	0.15
Sex, male	0.45 (0.13, 1.85)	0.30
HIV-positive	0.52 (0.17, 1.62)	0.26
Ever smoked^1^	1.16 (0.29, 4.62)	0.83
Poverty^2^	3.12 (0.87, 11.7)	0.08

*COPD* chronic obstructive pulmonary disease, *FEV*_1_/*FVC* < 0.7

^1^Current and former smokers combined

^2^Patients in the lowest socioeconomic position quintile vs. the patients in all other socioeconomic position quintiles

### Health-related QoL and socioeconomic position

The median SGRQ score was 7.8 (IQR 3.1–14.8) [15]. The median MHS score was 52.9 (IQR 47.8–57.9). Overall, 19% (18/95) of participants were in the first (poorest) relative socioeconomic position (SEP) while 34% (32/95) were in the highest relative SEP (Table 4).

**Table 4:** Summary of quality of life—MOS-HIV, SGRQ, and SEP

Quality of life measure	Median (IQR) or *N* (%)
MOS-HIV	
Physical health summary score	58.6 (52.0, 61.5)
Mental health summary score	52.9 (47.8, 57.9)
SGRQ	
Total score	7.8 (3.1, 14.8)
Median score for those with FEV_1_ ≥ 80% of predicted	4.9 (3.1, 10.5)
Median score for those with FEV_1_ 50–79% of predicted	9.4 (3.1, 19.4)
Median score for those with FEV_1_ < 49% of predicted	14.2 (9.4, 16.8)
SEP	
1—poorest	18 (19%)
2	10 (11%)
3	15 (16%)
4	20 (21%)
5—wealthiest	32 (34%)

*MOS-HIV* Medical Outcomes Survey HIV, SGRQ St. George’s Respiratory Questionnaire, *SEP* socioeconomic position

Bivariate analyses indicated that age, sex, HIV status, time since treatment completion (months), and poverty status were statistically significant predictors of COPD and were included in the multivariate logistic regression model, as shown in Table 3.

## Discussion

In this study of patients who completed treatment for pulmonary MDR TB at two teaching hospitals in Uganda, the prevalence of COPD was high, and patients reported poor mental and physical health. To our knowledge, this is the first study to evaluate lung function and QoL among patients completing MDR TB treatment in Uganda. Our results indicate that successful microbial cure of pulmonary MDR TB, while important, does not fully address the overall health impact of MDR TB. Pulmonary rehabilitation which has been shown to improve COPD symptoms in low resource settings like ours would be beneficial for patients completing MDR TB treatment [16].

The prevalence of COPD of 23% in our study is higher than the 16.2% prevalence reported in the general population of Uganda [4]. This is due to the consequences of MDR TB disease, which causes airflow obstruction and other lung function abnormalities [4, 17, 18]. It is also higher than the 16.7% prevalence of COPD reported by Byrne et al., among patients that had completed MDR TB treatment in Peru [19]. This difference is likely attributable to the fact that participants in their study were at least 3 years post-treatment*.* In contrast, Godoy et al., in Brazil, found the prevalence of COPD (FEV_1_/FVC < 70%) to be 39% among patients cured of MDR TB, which is higher than our study finding [20]. It is important to highlight that their study population constituted of only 18 participants who had a positive history of TB treatment before the MDR TB diagnosis and of whom 67% were former smokers (compared to 24% in our study). Although exposure to biomass fuels is a more prevalent risk factor for obstructive airway disease than cigarette smoking, the association between COPD and biomass fuel exposure is not well established in Uganda [4, 21]. A further evaluation of the primary etiologies of obstructive airway disease among previously treated MDR TB patients is warranted, especially given the increase in pollution and poor air quality in the urban areas of Uganda. Singla et al. found that 97.6% of patients who completed MDR TB treatment in India had abnormal pulmonary function tests, but they considered a higher cutoff of FEV_1_/FVC ratio of < 0.8, with a FVC 80% of the predicted value [2]. Additionally, they had a significantly smaller sample size of only 46 patients [2].

A low QoL in the mental and physical health domains among patients with MDR TB before, during, and after treatment has been demonstrated elsewhere in Pakistan and Namibia [22, 23]. Stigma, financial impoverishment, previous history of tuberculosis, and prolonged treatment before MDR TB diagnosis may be larger contributors to the low QoL [23–25].

In bivariate analyses, a history of smoking, unemployment, and low FEV_1_/FVC values were all found to be associated with lower mental and physical quality of life. However, due to the low sample size and subsequent lack of power, causation for poor mental and physical quality of life was unable to be determined.

MDR TB and its treatment have a catastrophic financial impact on patients [26], yet few studies have evaluated the SEP of patients after completion of treatment. van den Hof et al. found that the total costs for diagnosis and treatment of MDR TB represented 9–25 months of pre-treatment household income and up to 76% of patients reported job loss due to illness [27]. It is possible that the observation of worsening SEP over time since MDR TB treatment completion in our study is a result of the loss of income due to illness, incurred debt during treatment, and withdrawal of financial support upon completion of treatment all of which may trigger sale of household assets [28]. Participants with low QoL (MOS-HIV) scores are more likely to be unwell and less able to work. Those with a low QoL are more likely to have smoked, which would explain the association between low QOL, smoking, and its effect on mental health.

In our study, participants reported minimal impairment in respiratory health status, as assessed by the widely used St. George’s Respiratory Questionnaire. Byrne et al. also found that the frequency of respiratory symptoms such as wheezing, dyspnea, and phlegm were similar between controls that had never suffered TB and former MDR TB patients [19]. It has been observed that among MDR TB patients with COPD, the objective measurement of lung function does not correlate well with their subjective assessment of their symptoms [29]. This could explain the relatively normal SGRQ scores reported in our study.

Our study had some important limitations. One limitation of our study is that the cross-sectional nature of our evaluation limits the establishment of casualty. It is possible that study participants could have had COPD before acquiring MDR TB, given that COPD is a risk factor for MDR TB [30, 31]. Further, the use of mobile phone contacts during the sampling procedure may have introduced non-response bias from potential participants who did not own a mobile phone. Participants with mobile phones may have had a higher SEP score than non-users of mobile phones [32]. We did not control for other co-morbid conditions, other than HIV, yet these could affect the physical and mental health scores. Also, other common risk factors for COPD in Uganda such as exposure to biomass fuels were not adequately measured, and this can be done in a larger prospective study. The SGRQ was interviewer-administered due to language barriers, and this non-standard method of SGRQ administration could have affected the reporting of respiratory health status. The translation of the questionnaire into a local language such as *Luganda* would perhaps have yielded better discrimination between participants with and without COPD [33], but the *Luganda* validation of the SGRQ was not published until after our study was underway. Lastly, our sample size is small, so these results should be validated in larger studies.

## Conclusion

After MDR TB treatment, the prevalence of COPD is high, and patients report a low health-related QoL in the domains of mental and physical health. Our study highlights the need for long-term lung care and follow-up as part of routine MDR TB treatment. We recommend a prospective study with a larger sample size to evaluate the benefit of long-term pulmonary rehabilitation among patients who complete treatment for MDR TB.

## Supplementary information

**Additional file 1:** Multivariable Logistic Regression Parameter Estimates for COPD1 (Observed FEV1/FVC < LLN).

## Data Availability

The datasets used during the current study are available from the corresponding author on reasonable request.

## Abbreviations

MDR TB: Multi-drug-resistant tuberculosis
COPD: Chronic obstructive pulmonary disease
QOL: Quality of life
MOS-HIV: Medical Outcomes Survey for HIV
PHS: Physical health summary
MHS: Mental health summary
SEP: Socioeconomic position
SGRQ: St. George Respiratory Questionnaire
WHO: World Health Organization
LLN: Lower limit of normal
FEV_1_: Forced expiratory volume in 1 s
FVC: Forced vital capacity
