# Supplementary material for: Obstructive lung disease and quality of life after cure of multi-drug-resistant tuberculosis in Uganda: a cross-sectional study
Source: Trop Med Health. 2020 May 19;48:34. doi: 10.1186/s41182-020-00221-y (PMC7236316; doi:10.1186/s41182-020-00221-y)
Supplement: Supplementary file 1 — Additional file 1: Multivariable Logistic Regression Parameter Estimates for COPD1 (Observed FEV1/FVC < LLN). [file 41182_2020_221_MOESM1_ESM.pdf]

Appendix 1: Multivariable Logistic Regression Parameter Estimates for COPD[1] (Observed FEV1/FVC < LLN)

Outcome	Odds Ratios (95% Confidence Interval)	P-value
**COPD (Observed FEV1/FVC < LLN)**		
HIV-Positive	1.20 (0.93, 1.54)	0.16
Time Since Treatment Completion, years	0.96 (0.87, 1.05)	0.37
Ever Smoked[2]	0.68 (0.45, 1.04)	0.08
Poverty[3]	0.59 (0.29, 1.21)	0.15

[1]COPD = Chronic Obstructive Pulmonary Disease, here defined as an observed FEV1/FVC less than the lower limit of normal (LLN)

[2]Current and Former smokers combined

[3]Patients in the lowest socioeconomic position quintile vs. the patients in all other socioeconomic position quintiles
